# Biophysical Insights on the Enrichment of Cancer Cells from Whole Blood by (Affinity) Filtration

**DOI:** 10.1038/s41598-018-37541-3

**Published:** 2019-02-04

**Authors:** Marc Zinggeler, Thomas Brandstetter, Jürgen Rühe

**Affiliations:** Laboratory for Chemistry and Physics of Interfaces, Department of Microsystems Engineering (IMTEK), University of Freiburg, Georges-Koehler-Allee 103, 79110 Freiburg, Germany

## Abstract

Circulating tumor cells (CTCs) play a key role during the metastatic process of human cancers and their reliable detection and characterization could enable new and effective ways of cancer diagnosis, monitoring and treatment. However, due to their ultralow concentration in patient blood, the CTCs must first be enriched before such analysis can be performed. Classical microfiltration is an important and widely used method for the mechanical enrichment of CTCs. This method exploits that CTCs are generally larger than the accompanying blood cells, however, does not differentiate the cells in other ways. In an affinity filtration, selectivity is added by functionalizing the membrane with specific antibodies against a CTC-characteristic surface protein such as the epithelial cell adhesion molecule (EpCAM). A common shortcoming of both filtration approaches is that there is still a poor understanding of the enrichment process and the systems developed so far are frequently operated under non-optimized conditions. To address this, systematic filtration experiments are performed in this work using the EpCAM^+^ cell line MCF-7 as CTC-model and standard track-etched membranes modified with or without antibodies against EpCAM. The influences of the key filtration parameters time and applied pressure are studied and it is found that in all cases the extent of cell recovery is limited by a lysis process which occurs on the membrane surface. Counterintuitively, it is found that filtration at rather high pressures is advantageous to ensure high recovery rates. To describe the pressure-induced lysis process a biophysical model is developed. This model allows the determination of optimum filtration conditions to achieve both high cancer cell recovery and large blood sample throughput. It is demonstrated that this way practically 100% of spiked cancer cells can be recovered from milliliters of undiluted whole blood within seconds.

## Introduction

Cancer is a major cause of death and morbidity worldwide^1^. In Europe alone, there were an estimated 3.45 million new cases of cancer and 1.75 million deaths from cancer in 2012^2^. Including the most common types of cancer like breast, colorectal, prostate and lung cancer, carcinomas represent by far the most abundant class of tumors. Carcinomas are solid tumors derived from epithelial tissue and most deaths from this class of tumors are caused by the haematogenous spread of cancer cells from the primary tumor into distant organs and their subsequent growth to metastases^3,4^. In this complex process Circulating tumor cells (CTCs) were found to play a key role in this complex process and their reliable detection and characterization could enable new and effective strategies for cancer diagnosis, monitoring and treatment^4–7^. However, the analysis of CTCs still represents a strong analytical challenge due to their ultra-low abundance of a few cells per mL of blood, while they are at the same time covered by billions of blood cells^7–9^.

The reliable analysis of CTCs using standard microscopic methods like immunocytochemistry (ICC), therefore, strongly depends on an efficient CTC-enrichment step. It was discovered already in 1964 by S. H. Seal that CTCs can be isolated from whole blood by classical dead-end microfiltration due to their larger size and lower deformability compared to normal blood cells^10^. Compared to other methods of cell separation, filtration is of special interest due to its high efficiency, cost-effectiveness, handling-simplicity, good compatibility with downstream analysis of cells and high sample throughput^7,11–15^. Filtration studies performed so far, mainly focused on different membrane geometries^16–25^, or were concerned with the comparison of filtration to other methods of enrichment^26,27^. With the aim to enhance the performance of classical filtration, the concept of affinity filtration was introduced^28^. The key concept of the system is to enrich CTCs based on a combination of mechanical and molecular interaction with the membrane and therefore include a second level of selectivity through immobilization of CTC-selective capture molecules (e.g., anti-EpCAM) on the membrane. This concept has in the meantime been studied by other investigators and a beneficial effect of the affinity filtration has been reported^29^. However, there has not yet been a systematic study on the influence of the filtration parameters on the enrichment process. This leaves us with a still poor understanding of the filtration process and the systems developed so far operate under non-optimized conditions.

The aim of this work is to address these shortcomings and acquire an in-depth understanding of the filtration process. To this end, we generate CTC-selective affinity membranes from track-etched filters with 8 µm size cut-off, which are widely used for this application, and use a hydrostatic filtration system that enables exact control over the filtration pressure. In systematic filtration experiments using the EpCAM^+^ cell line MCF-7 as a CTC-model, the influences of the identified key filtration parameters time and pressure on the recovery of the system are studied and a model to describe the filtration process is developed. Using optimized filtration conditions, the performance of the system is studied in blood spiking experiments and compared to classical filtration.

## Results and Discussion

### Preparation and characterization of affinity membranes

**Figure 1:**
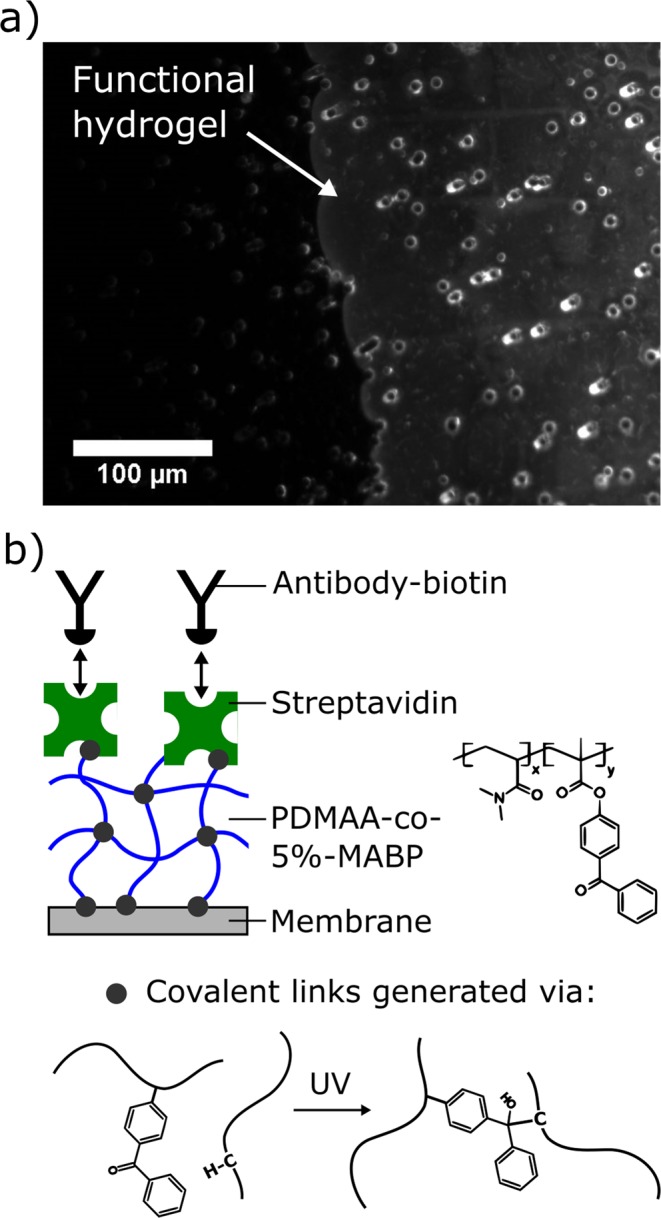
Generated affinity membranes. (**a**) Fluorescence micrograph of the hydrogel coated track-etched membrane. The hydrogel coating was stained by immobilization of fluorescently labeled streptavidin. (**b**) Schematic depiction of the functional hydrogel coating.

One of the most widely used commercially available membranes for the enrichment of CTCs from blood consist of track-etched polycarbonate (PCTE) with a size cut-off of 8 µm^19,30,31^. These cost-effective materials are produced on large scale by ion-bombardment followed by etching and are characterized by randomly distributed circular pores with controlled diameter. Due to their random pore arrangement the porosity of these materials is typically limited to <10% to avoid an excessive overlapping of pores. The selected product for this study was an 8 µm hydrophilic PCTE with a thickness of 7 µm and a porosity of 5% (Sterlitech corp.). For this membrane an average pore diameter of 7.2 µm was measured optically. To transform these membranes into cell-selective affinity membranes, we developed a surface-engineering process based on a photo-immobilization process. To this end, a thin layer of a photo-reactive copolymer (poly(N,N-dimethyl acrylamide-stat-5% methacryloyloxy benzophenone) is deposited on the membrane and irradiated by UV light. This process leads to the generation of covalently cross-linked and surface-attached polymer networks on the polymeric substrates by C,H-insertion reactions triggered by the UV-exposure^32–34^. For modification of the PCTE the polymer was deposited by dip coating from ethanolic solution followed by a first UV-irradiation step at 365 nm. The energy dose for this first UV-treatment was set to 1 J/cm² to form a stable network (i.e., 100% gel content) using only a small fraction of the benzophenone groups (around 15%) contained in the polymer layer. The coated PCTE was then saturated with streptavidin followed by a second UV-treatment (254 nm, 0.5 J/cm²) to complete the photochemical reactions in the layer. During this second illumination step covalent immobilization of streptavidin molecules on the surface can be achieved. Using fluorescently labeled streptavidin it was found that the PCTE was homogeneously coated with the functional hydrogel layer including the inner surfaces of the pores (Fig. 1a). To determine the hydrogel layer thickness inside the pores, the fluidic membrane resistance was measured before and after the modification procedure. For the filtration experiments not so much the mass of the pore coating itself, but the reduction of the pore diameter induced by the swollen coating is an important parameter. From the membrane resistance measurements, it was concluded that the average pore size of the membrane was reduced from 7.2 µm to 6.7 µm indicating a swollen hydrogel layer thickness of 250 nm. This reduction in pore size is considered non-critical, since an optimal pore size between 6 and 7.5 µm has been reported for the enrichment of CTCs^16^. The formed networks swell strongly (approx. by factor 2.5) when subjected to an aqueous environment and the so obtained hydrogel layers were found to effectively protect surfaces against non-specific protein adsorption and cell adhesion (i.e., “anti-biofouling” properties)^35^.

The surface concentration of the immobilized streptavidin was measured to be around 30 nmol/m². This value is comparable to the theoretical surface concentration of 25 nmol/m² for a streptavidin monolayer based on a unit-cell size of 8.2 nm^36^. If lower streptavidin concentrations are desired, this can readily be achieved in a controlled fashion by increasing the illumination dose during the first UV-treatment as it has been shown previously^28^.

The hydrogel coated membrane can be functionalized by simple incubation with any desired biotinylated capture molecule exploiting the extraordinary strong biotin-streptavidin interaction (Fig. 1b). A coupling yield of around 30% (i.e., fraction of streptavidin molecules undergoing biotin coupling) was measured for the immobilized streptavidin using a bi-functional DNA probe carrying both a biotin and a fluorescence label. This value is comparable to the reported activities of 27–37% which were measured for antibodies after the covalent immobilization using different wet chemical approaches^37^. Additionally, it was found that the immobilized streptavidin that survived the immobilization process is fairly stable and >80% of retained activity was measured after 8 months dry storage at ambient conditions. To complete the generated hydrogel surface for an application in CTC-enrichment a biotinylated anti-EpCAM antibody was used. The selectivity of the antibody functionalized surface was studied both on the level of proteins and cells and the results were published previously^28^. In both cases the surface showed excellent selectivity enabling both a strong interaction with EpCAM positive cancer cells and at the same time effectively preventing the non-specific adhesion of blood cells or proteins.

### Description of the used CTC-model and filtration setup

**Figure 2:**
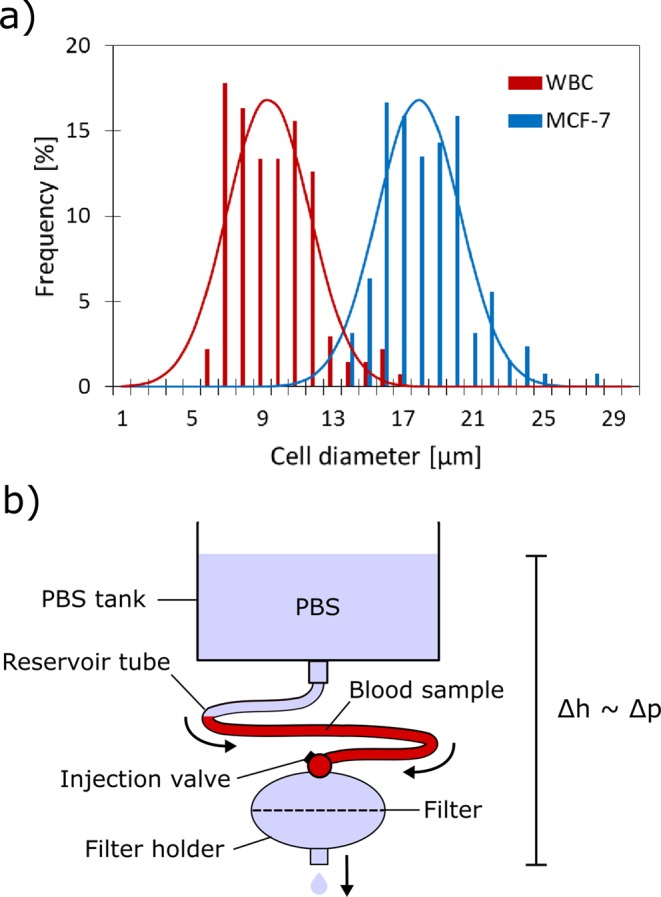
Used CTC-model and filtration setup. (**a**) Measured size distributions (histograms) and fitted normal distribution functions (lines) for WBCs and MCF-7 cells which serve as a model for epithelial CTCs. (**b**) Schematic depiction of the hydrostatic filtration setup.

The state-of-the-art method to measure the enrichment performance of novel CTC-isolation technologies is to use well-known cancer cell lines as CTC-models. In such experiments a known number of these cancer cells are spiked into a defined blood volume which is subsequently processed using the method under investigation. This allows an accurate measurement of the recovery of the system, which is considered the most important performance indicator, and enables the reliable comparison with other systems. Additional parameters which should be determined are throughput (i.e., processed blood volume per time) and purity (i.e., number of captured CTCs compared to the number of erroneously captured blood cells). For this study it was decided to use the metastatic breast cancer cell line MCF-7 as a model for epithelial CTCs. This EpCAM^+^ cell line is one of the most widely used for this purpose and shows, comparable to CTCs, an overlapping size distribution profile with WBCs (Fig. 2a). The optically determined median cell sizes for MCF-7 and WBCs were 17.7 µm and 9.1 µm respectively.

For the controlled isolation of cancer cells from blood a hydrostatic filtration setup was constructed (Fig. 2b). The setup consisted of a large PBS reservoir which was connected to the filter holder using flexible tubing. Between this tubing and the filter holder a valve was mounted to enable the simple injection of a blood sample. Once injected into this tubing (further referred to as “reservoir tube”) the blood sample can be filtered with a highly controlled filtration pressure given by the hydrostatic pressure. Due to the large dimensions of the PBS container this pressure can be assumed to remain constant over typical experimental time scales. Additional benefits of this system are that there is no need for a pump and that sample processing and washing can be achieved in a single continuous filtration step.

### Time and pressure dependency of the enrichment process

**Figure 3:**
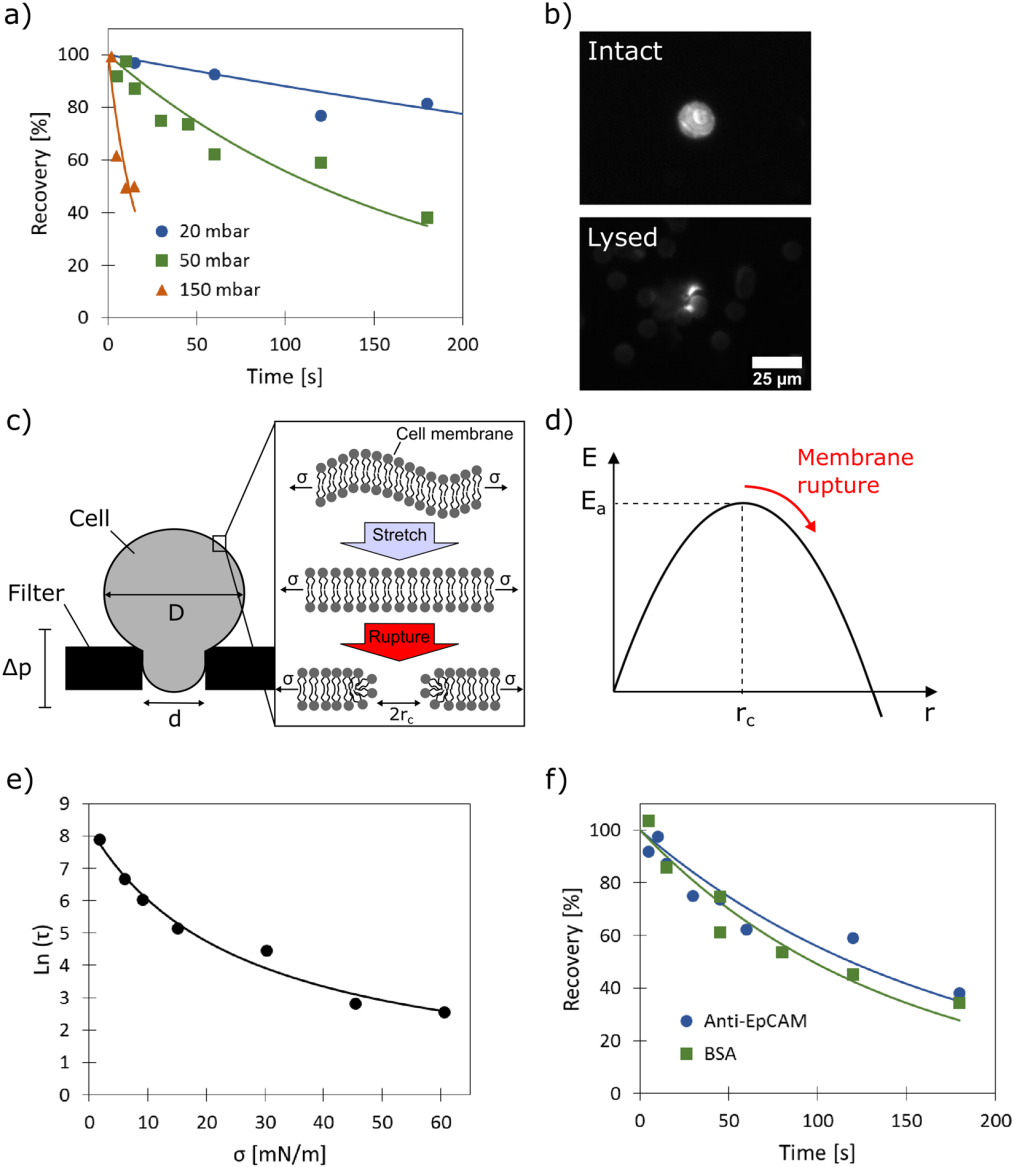
Time and pressure dependency of the enrichment process due to cell lysis. (**a**) Measured recoveries (symbols) as a function of filtration time for different filtration pressures using anti-EpCAM modified PCTE. Solid lines provide best fits using Eq. (1). (**b**) Fluorescent micrographs of an intact and lysed cell (cell residues) on a membrane surface. The shared scale bar is shown in the bottom image. (**c**) Schematic depiction of a cell penetrating a pore due to the exerted pressure difference across the membrane. (**d**) Theoretical energy diagram of the membrane rupture process based on Eq. (3). (**e**) Logarithmic plot of experimentally determined time constants (black dots) as a function of the exerted tension (σ) induced by different filtration pressures. The solid line provides a best fit using Eq. (5). (**f**) Measured MCF-7 recoveries (symbols) as a function of filtration time at a filtration pressure of 50 mbar for anti-EpCAM and BSA functionalized membranes. The solid lines in respective colors provide best fits using Eq. (1).

To study the influence of the filtration time and pressure, which were identified as the two key parameters, on the recovery of MCF-7 cells, model experiments in PBS were conducted. To this end, 1000 (±50) pre-stained MCF-7 cells were spiked to 1 ml PBS. The sample was then injected into the reservoir tube, followed by filtration under controlled conditions. Due to the small injection volume the time required by the cells to reach the filter surface is negligible compared to the studied filtration times. After filtration the captured cells on the filter were counted under a fluorescence microscope to determine the recovery. The recovery values for PCTE membranes functionalized with anti-EpCAM were studied for filtration pressures ranging between 6 and 200 mbar (three examples are shown in Fig. 3a). In all cases it was found that the recovery (R) dropped exponentially over time (t) well described by the following equation:

1 $$R(t)={R}_{0}{e}^{-t/\tau }$$

where R_0_ represents the perfect recovery (i.e., 1 or 100%) and τ represents a characteristic time constant. This behavior indicates that a steady-state will never be reached where cells will reside stably on the filter surface after capture. This behavior is at first view quite surprising for an affinity filtration, because the adhesive interaction between the strongly EpCAM expressing MCF-7 cells (an EpCAM surface concentration (C_c_) of 2.8·10^14^ molecules/m^2^ has been reported^38^) and the capture surface should effectively prevent cell losses caused by squeezing of cells through the pores. Based on the reported binding strength (f_c_) between EpCAM and an anti-EpCAM antibody of 6.7·10^−11^ N^38^, and a maximum interaction area (A_c_) of 1.5·10^−10^ m^2^ (i.e., the inner wall of the pore) we calculate an adhesive strength (F_A_) of 2.8·10^−6^ N (F_A_ = f_c_ * A_c_ * C_c_, according to^38^). This corresponds to a pressure difference of 780 mbar acting on the cross-sectional area of the pore. So even if a much lower contact area is considered or not all EpCAM molecules have yet bound to an antibody (the antibody density on the capture surface is in the order of 5.4·10^15^ m^−2^) one would expect to observe permanently captured cells at least in low pressure experiments. Based on this inconsistency, it must be suspected that there is an additional loss mechanism involved that is responsible for the clearance of stably captured cells from the membrane. To investigate this further we analyzed the filtrates of several experiments using PCTE with 3 µm pores and found that in all cases significant numbers of cells completely disappeared from the system, strongly indicating cell lysis. Upon careful examination of the capture membrane, cell residues were clearly observed at some pore locations, which support this hypothesis (Fig. 3b). Cell lysis was already suspected during filtration by several investigators^16,24,39^, however, no model for this behavior has so far been provided. It has for example been speculated that the lysis may preferentially occur immediately upon initial collision of the cell with the pore or that there exist a critical pressure a cell can resist^24^. However, this is in strong contradiction to our results, which clearly show that cells can successfully be captured even at extraordinary high filtration pressures of up to 200 mbar when the filtration time is held short enough.

To better understand this lysis mechanism theoretical frameworks derived from micropipette aspiration studies were applied and it was found that such models describe our experimental results quite well. This is because once a cancer cell is placed on a pore and some pressure is applied it almost perfectly resembles the situation in a typical micropipette aspiration experiment. This resemblance was already noticed in a short publication by Lu *et al*.^40^, where the viability of cancer cells on a membrane has been studied by life-dead staining. However, no correlation has been made between cancer cell recovery and the filtration conditions.

The filtration process can be described as follows:

Upon the arrival of a cell on a pore a tight seal between the cell and the filter membrane is created. In this state the cell experiences the pressure differential across the membrane (i.e., the hydrostatic pressure) for a given time period (Fig. 3c). The pressure difference (Δp) will induce a tension (σ) in the cell membrane according to^41^:

2 $$\sigma =\frac{\Delta p\cdot d}{4(1-d/D)}$$

where d is the pore diameter and D is the cell diameter. Upon exertion of this tension the cell membrane will be stretched causing the cell to penetrate deeper into the pore. It was calculated that for an average (17.7 µm) MCF-7 cell to pass through an average (6.7 µm) pore it has to undergo a deformation leading to an increase in surface area of around 25%. The phospholipid bilayer itself, which is forming the cell membrane is rather inelastic and critical areal strains on the order of 2–4% were reported^42,43^. Accordingly, such large deformations can only be accomplished by a cell in a non-lethal way (i.e., without undergoing cell membrane rupture) through the unfolding of membrane wrinkles leading to an apparent increase in surface area. Once the membrane of a cell has been stretched out completely, it cannot penetrate any deeper into the pore. In the case of affinity filtration such a state may be reached earlier due to the adhesive interaction with the antibodies presented on the pore walls. However, when the membrane is subjected to such tension, nanometer sized pores will be formed by thermal activation/energy fluctuations in the cell membrane.

These pores can open and possibly close again (healing of the membrane), However, when enough energy is put into the system, large and more stable pores are formed that eventually lead to membrane rupture. The energy of a pore formed in the lipid membrane (E) can be described by the following equation^44^:

3 $$E=2\pi r\gamma -\pi {r}^{2}(\sigma +c)$$

where the first term is the edge energy of the pore having a radius r and line tension γ. The second term describes the energy released during the nanopore opening as it reduces the membrane tension. It includes also an energy term c which accounts for the energy changes due to the rearrangement of the molecular components of the membrane and possibly surface charges^40,45^. In the present study this term serves additionally as a correction term to account for cell losses not connected to lysis (i.e., squeezing losses). According to Eq. (3), E follows a parabolic profile when plotted against r with a maximum E_a_ (dE/dr = 0) which represents the activation energy at the critical radius r_c_ = γ/(σ + c) (Fig. 3d). Inserting r_c_ into Eq. (3) one obtains:

4 $${E}_{a}=\frac{\pi {\gamma }^{2}}{(\sigma +c)}$$

When this critical radius r_c_ is overcome, the cell membrane will rupture causing cell lysis. As the kinetics of cell lysis is thus connected to this activation energy, the lysis process is a purely stochastic process which explains why the experimental data is well described by Eq. (1).

To further describe the pressure (i.e., tension) dependency of the determined time constants the Arrhenius equation is used:

5 $$\tau =\frac{1}{k} \sim {e}^{(\frac{{E}_{a}}{{k}_{B}T})} \sim {e}^{(\frac{\pi {\gamma }^{2}}{{k}_{B}T(\sigma +c)})}$$

with k as the rate constant, k_B_ as the Boltzmann constant and T as the absolute temperature. Eq. (5) was then used to fit the experimentally determined time constants for the different tensions (induced by the different filtration pressures) and the result is shown in Fig. 3e. It was found that the model describes the data well, offering us a valuable tool to find the optimum filtration pressure.

As in all cases the cells are primarily “locked” in their position at the small pores due their limited deformability, the recovery is mainly determined by how many cells survive cell lysis. Additional binding of the cells through specific antibodies was therefore found to have practically no influence on the recovery of the system (Fig. 3f). The affinity effect, i.e., an effect by additional cell binding, may be more pronounced for membranes containing larger pores. However, in this case one would risk losing smaller or EpCAM-low target cells.

### Finding the optimum filtration pressure

**Figure 4:**
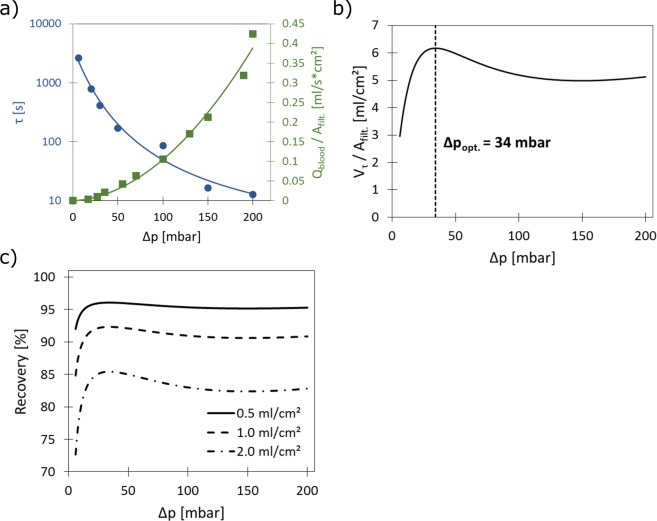
Prediction of optimal filtration conditions. (**a**) Measured (symbols) and calculated (solid lines) recovery time constants (blue, left y-axis) and blood flux (green, right y-axis) as a function of filtration pressure. (**b**) Calculated characteristic blood volumes per area for different filtration pressures obtained by multiplication of the two functions provided in graph (a). An optimum is observed at 34 mbar enabling the highest volume throughput. (**c**) Predicted recoveries as a function of filtration pressure for different blood sample throughputs.

The optimum filtration pressure is defined in the following as the pressure which provides the best combination of high recovery and large sample throughput. To find this value the characteristic time constants determined in the previous chapter (Fig. 3e) for each pressure need to be compared to the individual blood flow values caused by the application of these pressures. The required blood flow characteristics were measured for an average whole blood sample obtained by pooling 10 compatible samples from healthy donors. The data of the blood flux, i.e., the blood flow normalized to the filter area (Q_blood_/A_filt_), as function of the applied pressure is shown in Fig. 4a and reflects the well-known shear-thinning behavior of blood. To interpolate between the data points a shear-thinning model published by Byun *et al*.^46^. was used, which was found to describe the experimental data well. The provided blood flow characteristics were normalized for the filter area because this is considered an important parameter for the scaling behavior of the system. It is important to note that these characteristics can also be strongly influenced by the porosity of the membrane.

An important parameter in any filtration experiment is the volume of analyte which can processed in a given filtration time. How fast the filtration process is, however, has a strong influence on the recovery. In case τ is used as the filtration time, identical recoveries are obtained for each filtration pressure. The sample throughput will vary with the applied pressure and is described by a characteristic blood volume (V_τ_) for each pressure that is processed within the filtration time τ. However, this volume must be normalized to the filter area (A_filt._). These values of V_τ_/A_filt_ as a function of the filtration pressure are obtained by multiplying the two functions shown in Fig. 4a and the results are shown in Fig. 4b. A maximum is observed at a pressure of 34 mbar, which indicates the presence of an optimum filtration pressure for the membranes used in this study (i.e., at the given pore geometry).

To calculate the recovery that can be expected for this scenario, i.e., when τ is used as the filtration time, one may be tempted to use Eq. (1). However, this is incorrect since cancer cells contained in a blood sample will arrive throughout the filtration process on the membrane and therefore will experience different times sitting on the pores on the membrane. To calculate the recovery R(t) in this case, two integrals need to be compared: The first integral represents the number of captured cells at time t and is obtained by integrating Eq. (1) (with R_0_ = 1) from t = 0 to t. The second integral represents the total number of cells in the system and is obtained by integrating the function R(t) = 1 from t = 0 to t. After integration and rearrangement one obtains:

6 $$R(t)=\frac{{\int }_{0}^{t}{e}^{-t/\tau }dt}{{\int }_{0}^{t}1\,dt}=\frac{\tau }{t}-\frac{\tau }{t}{e}^{-t/\tau }$$

Using Eq. (6) and the determined τ values, the expected recoveries for any desired filtration conditions can be predicted. For the situation illustrated in Fig. 4b (i.e., t = τ) a recovery of 63% is calculated.

A far more common situation in diagnostics is that a given sample volume i.e., a standard size blood sample, needs to be processed. As an example for this situation, predicted recoveries for three different sample volumes per area as a function of the filtration pressure are shown in Fig. 4c. The plot shows what recovery can be expected as a function of pressure for a filter with a given filter area and different blood volumes or vice versa how large the filter area needs to be selected if a certain recovery is targeted. To achieve high recoveries (>95%) the sample volume per filter area needs to be kept low (≤0.5 ml/cm^2^) and sufficient pressure should be provided (≥34 mbar). This means that a sufficient filter size needs to be selected to process a certain blood volume with high recovery (for example 15 cm² for 7.5 ml). In this case very short filtration times are used (for example 40 s for 34 mbar). Interestingly, the curve almost plateaus at higher pressures. This demonstrates that it is quite advantageous to use filtration conditions in which a rather high pressure difference is applied. Lower pressures, in contrast to a straight forward intuition, are unfavorable due to the very low blood flows which in turn cause extraordinary long residence time of the cells on the membrane. Such long residence times induce significant cell lyses. It can be seen in Fig. 4c that all pressure differences below 30 mbar lead to a significant loss of cells independent of the filter to volume ratio.

In addition to the lysis problem it is noted that at very low pressure (6 mbar) in some cases clogging of the filter was observed, which was never observed for larger blood filtration pressures. In the case larger sample volumes per area are used (i.e., longer filtration times) working at the optimum filtration pressure (34 mbar in this case) is favorable.

### Blood spiking experiments

**Figure 5:**
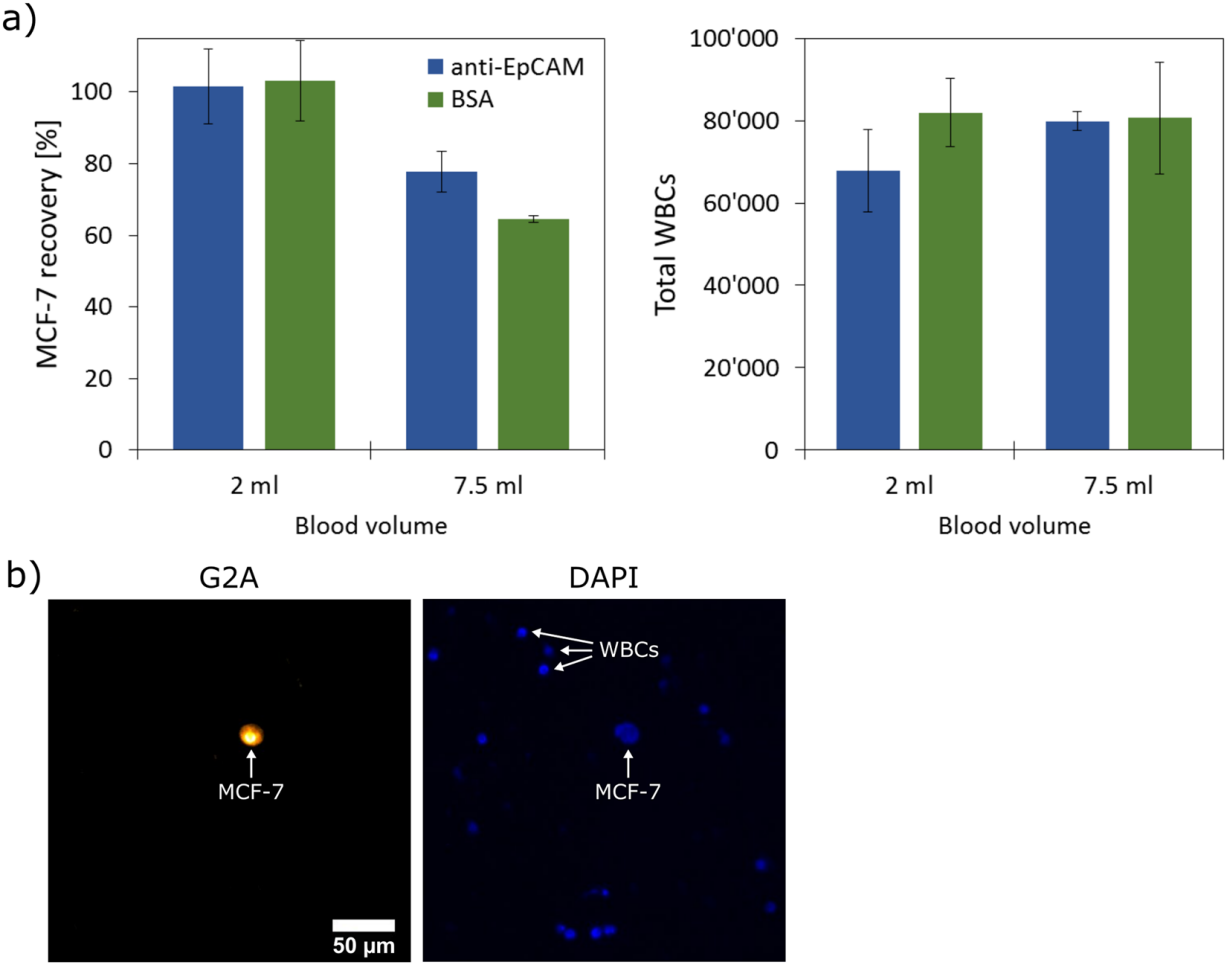
Results from the enrichment of spiked MCF-7 cells from non-diluted whole blood (volume: 2 or 7.5 ml) by filtration at 34 mbar using filters with an area of 3.5 cm² and different functionalization (anti-EpCAM or BSA). (**a**) The measured MCF-7 recoveries (left) and number of WBCs (right) are shown (n = 3, mean values and standard deviations are shown). (**b**) Fluorescence micrographs of a filter section recorded in G2A- (cytosol staining of MCF-7) and DAPI-channel (nuclei staining of MCF-7 and WBCs). The shared scale bar is shown in the left image.

To study the enrichment of cancer cells from whole blood using the determined optimum filtration pressure of 34 mbar, blood spiking experiments were conducted. To this, 1000 (±50) pre-stained MCF-7 cells spiked into 2 or 7.5 ml pooled whole blood served as the analyte. These samples were injected into the reservoir tube of the filtration setup and then filtered within 40 and 120 s respectively through modified PCTE with an effective filtration area of 3.5 cm². Within these times the complete sample volumes were processed including a short washing period. Based on Eq. (6) and the determined τ-value of 394 s for 34 mbar, 95% and 86% recovery are expected for an affinity filtration for the two studied cases. The measured recoveries for PCTE modified with anti-EpCAM (affinity filtration) and BSA (classical filtration) are shown in Fig. 5a. Additionally, the number of captured WBCs for the different experiments is provided, which was determined by counting nucleated cells on the membrane lacking the MCF-7 cytosol pre-staining (Fig. 5b). For the low volume example no difference was observed between the two membranes and in both cases >95% recovery was obtained. In the large volume example the recovery of the control membrane dropped to 65% while the affinity membrane retained 78%. The measured recovery values for the affinity filtration (>95%, 78%) are in close agreement to the calculated predictions (95%, 86%) and the small gain observed for the affinity membrane for larger sample volumes (i.e., longer filtration time) is in good agreement to the results discussed in Fig. 3f.

The achieved combination of high recovery (>95%) and large sample throughput (2 ml of undiluted whole blood processed within 40 s) is very advantageous for CTC isolation and compares very favorably with the literature^29^. When choosing suitable filtration conditions, one would intuitively think that filtration at very low pressures is advantageous as it is gentle to the cells and ensures high survival rates; however, this is not completely true. One misses out in these considerations that at very low pressures also very long filtration times are required. As a consequence the cells experience this stress for very long times which leads to significant cell lysis and to a strongly reduced recovery rate. Within some limits the survival rate of the cells is higher when they endure a stronger stress for a short time than being exposed to a moderate stress for a very long time.

The number of contaminating WBCs on the filters was in all cases at around 80000 cells (covering roughly 15% of the pores), which is comparable to values reported in the literature for PCTE^16^. Interestingly, this number was practically independent from the sample volume indicating that this number is mainly governed by traps (not completely etched pores or pore overlaps) on the membrane. Once these traps are filled with cells, the number of captured WBCs plateaus, which explains the fact that membrane clogging was never observed at 34 mbar. The deposited WBCs on the membranes were found to be well distributed and separated from the cancer cells, which enables a straight-forward identification and analysis of the captured cells without any need for further purification using widely used microscopic methods like ICC. For such analysis affinity filtration does not offer an advantage over classical filtration. However, many molecular down-stream analysis methods such as sequencing strongly benefit from higher CTC purities. To achieve this, it is noted that affinity membranes offer the unique advantage to remove erroneously captured leukocytes after the enrichment e.g., by backwashing while surface bound CTCs are retained. We will elaborate further on this aspect in a follow-up communication.

At this point it should be noted that the focus of the present work was to study the biophysics of CTC isolation by affinity filtration and not to develop a diagnostic tool. Accordingly, we used full blood samples and added CTC by spiking, so that we know exactly how many cells are contained in the sample. Furthermore it should be noted, that in this study the lowest cell concentration analyzed was around 130 cells/ml (i.e., 1000 in a typical blood sample of 7.5 ml), which is in patient samples only encountered in some cases where metastatic activity is high. However, when much lower cell numbers are envisaged, it is difficult to discuss smaller differences in the recovery rate, especially when the recovery rate is high. In such cases even one cell more or less would make a big difference in the numerical values. For example, in cases of 10 cells per sample or less, it will be difficult to differentiate between recovery rates of 80–95%. However, such differences are needed here for the development of the model. At the moment we study the technique at cell counts of 10 or even less cells per ml and move to patient samples with an a priori unknown cell count for a diagnostic tool development.

## Conclusions

During the enrichment of cancer cells by filtration using track-etched membranes, filtration time and applied pressure are the two key parameters which need to be controlled to reduce cell lysis occurring on the membrane surface and thus to ensure high capture efficiency and recovery of the desired cells. One would intuitively think that the lowest filtration pressures represent the mildest conditions and lead to the highest cell recoveries. However, the time required for filtration, i.e., the time during which the cells are exposed to a certain pressure is also an important factor. For low applied pressures of only a few millibars the times required for filtration become long and significant lysis occurs. Consequently, such conditions lead to significant loss of cells and the use of such low pressures must be avoided. High recoveries of practically 100% can be achieved using quite high filtration pressures, even of up to 200 mbar provided that the filtration time is held short enough. When an appropriate membrane, i.e., a membrane with a suitable porosity and size is taken, such conditions allow the processing of several milliliters of undiluted whole blood within less than 1 minute. Because the enrichment process is essentially governed by the physics, such performances can be achieved independent of membrane functionalization. Although, the purity of the isolated cells is sufficient to enable direct microscopic analysis, purity increases could have important implications for many molecular down-stream analytical approaches. To achieve this, affinity membranes are expected to play a key role as more effective washing procedures (e.g. backwashing) can be applied by selectively holding back surface bound CTCs. Further purity gains are expected as membranes containing a smaller number of defects and membranes with a tighter control of the pore diameter are employed. We are currently working along these lines.

## Materials and Methods

### Modification of PCTE

Poly(N,N-dimethyl acrylamide-stat-5% methacryloyloxy benzophenone was synthesized as described elsewhere^33^. PCTE (25 mm diameter, PCT8025100, Sterlitech) was dip-coated (withdrawal speed: 50 mm/min) with polymer solution (5 mg/ml in EtOH) followed by a first UV treatment (365 nm, 1 J/cm²). Membranes were washed by filtration with EtOH. Streptavidin (6073.3, Carl Roth) was deposited onto the membranes by drop coating from aqueous solution containing 1 mg/ml protein followed by drying at ambient conditions. Membranes were again illuminated with UV-light (254 nm, 0.5 J/cm²) before final washing with water by filtration. Membrane functionalization with biotinylated BSA (A8549, Sigma-Aldrich) or anti-EpCAM (BAF960, R&D Systems) was performed directly in the filter holder (Swinnex) by incubation with PBS solutions containing 1 µg/ml protein for 1 h prior to use.

### Characterization of modified PCTE

The filtration resistance of the membranes before and after modification was determined by measuring the steady-state water flow rate through the membranes at a constant hydrostatic pressure. Based on the measured resistances the pore sizes were calculated using a filtration model published elsewhere^47^. The streptavidin surface concentration was determined by fluorescence measurement of immobilized streptavidin-Cy5 (PA45001, GE Healthcare) in a standard fluorescence reader. The obtained fluorescence intensity was transformed to a surface concentration using a calibration curve. The same methodology was used to determine the coupling yield to biotin-DNA-Cy5 (custom made, TIB Molbiol) and the stability (coupling yield over time) of immobilized streptavidin.

### Construction of filtration setup

A plastic bottle with ID 8.5 cm was used as PBS tank which was connected to a Tygon reservoir tube with ID 3.17 mm. The length of the tube was adjusted according to the required hydrostatic pressure. As injection valve a standard infusion 3-gang manifold (Discofix, B. Braun) was used.

### Cell culture and staining

MCF-7 cells were cultured in RPMI 1640 medium (P04-17500, PAN Biotech) containing 10% FBS (10270-106, Thermo Fisher), 3 mM L-Glutamine (25030-081, Thermo Fisher) and 10 µg/ml insulin (11376497001 ROCHE, Sigma-Aldrich) in 5% CO_2_ at 37 °C. Cells were harvested by treatment with 0.25% (w/v) trypsin (25200-072, Thermo Fisher) when 70–80% confluency were reached. Cells were stained in suspension by incubation with 5 µM Cell Tracker Orange (C34551, Life technologies) solution in PBS for 30 min. The concentration of stained cells in suspension was determined using a hemocytometer (DHC-N01, NanoEnTek, CV: 5%) and was typically between 0.5–0.8 M cells/ml.

### Filtration experiments

The calculated suspension volume containing 1000 (±50) stained MCF-7 cells was spiked into a defined volume of PBS or pooled EDTA blood from 10 healthy donors with compatible blood groups. Blood samples were obtained from the University Medical Center Freiburg and informed consent has been obtained. The experimental protocol was approved by the ethics commission of the University of Freiburg with the number 308/15 and all methods were performed in accordance with the relevant guidelines and regulations. Spiked samples were injected into the reservoir tube followed by filtration with controlled pressure and time. The isolated cells were fixed to the membrane by filtering 1. 70% EtOH and 2. 100% EtOH at 6 mbar for 5 min each. For nuclei staining the membranes were “cover-slipped” with 1 µg/ml Hoechst 33342 (H1399, Thermo Fisher) solution in PBS. Cells were then counted under a fluorescence microscope (Nikon Eclipse TS100) using a counting grid (MCF-7: Cell Tracker^+^, Hoechst^+^; WBC: Cell Tracker^-^, Hoechst^+^). The number of total WBCs on the membrane was calculated from an average coverage measured at 10 random locations. Blood flow characteristics were measured using a syringe pump (540061, TSE Systems) and a pressure sensor (PCE-910, PCE-Instruments). Filtration experiments in whole blood were directly performed using the modified PCTE with a diameter of 25 mm. For the filtration experiments in PBS smaller membrane pieces with 13 mm diameter were punched out from the larger filters. Data analysis was performed on Excel (2010) using built-in data analysis features.

## Data Availability

The data that support the findings of this study are available from the corresponding author, J.R., upon request.
